# Pretreatment with Mangafodipir Improves Liver Graft Tolerance to Ischemia/Reperfusion Injury in Rat

**DOI:** 10.1371/journal.pone.0050235

**Published:** 2012-11-30

**Authors:** Ismail Ben Mosbah, Yann Mouchel, Julie Pajaud, Catherine Ribault, Catherine Lucas, Alexis Laurent, Karim Boudjema, Fabrice Morel, Anne Corlu, Philippe Compagnon

**Affiliations:** 1 Inserm, UMR991, “Foie, Métabolismes et Cancer,” CHU Pontchaillou, Rennes, France; 2 Université de Rennes 1, Rennes, France; 3 Laboratoire de Biochimie Générale et Enzymologie, CHU Pontchaillou, Rennes, France; 4 Service de Chirurgie Digestive et Hépatobiliaire-Transplantation hépatique, CHU Henri Mondor, AP-HP, Créteil, France; 5 Inserm, UMR955,- IMRB Université Paris Est, Créteil, France; 6 Service de Chirurgie Hépatobiliaire et Digestive, Hôpital Pontchaillou, Rennes, France; University of Colorado, United States of America

## Abstract

Ischemia/reperfusion injury occurring during liver transplantation is mainly due to the generation of reactive oxygen species (ROS) upon revascularization. Thus, delivery of antioxidant enzymes might reduce the deleterious effects of ROS and improve liver graft initial function. Mangafodipir trisodium (MnDPDP), a contrast agent currently used in magnetic resonance imaging of the liver, has been shown to be endowed with powerful antioxidant properties. We hypothesized that MnDPDP could have a protective effect against liver ischemia reperfusion injury when administrated to the donor prior to harvesting. Livers from Sprague Dawley rats pretreated or not with MnDPDP were harvested and subsequently preserved for 24 h in Celsior® solution at 4°C. Organs were then perfused *ex vivo* for 120 min at 37°C with Krebs Henseleit solution. In MnDPDP (5 µmol/kg) group, we observed that ATP content was significantly higher at the end of the cold preservation period relative to untreated group. After reperfusion, livers from MnDPDP-treated rats showed better tissue integrity, less hepatocellular and endothelial cell injury. This was accompanied by larger amounts of bile production and higher ATP recovery as compared to untreated livers. The protective effect of MnDPDP was associated with a significant decrease of lipid peroxidation, mitochondrial damage, and apoptosis. Interestingly, MnDPDP-pretreated livers exhibited activation of Nfr2 and HIF-1α pathways resulting in a higher catalase and HO-1 activities. MnDPDP also increased total nitric oxide (NO) production which derived from higher expression of constitutive NO synthase and lower expression of inducible NO synthase. In conclusion, our results show that donor pretreatment with MnDPDP protects the rat liver graft from cold ischemia/reperfusion injury and demonstrate for the first time the potential interest of this molecule in the field of organ preservation. Since MnDPDP is safely used in liver imaging, this preservation strategy holds great promise for translation to clinical liver transplantation.

## Introduction

Liver transplantation has been established as the most effective and durable therapy for end-stage liver disease [1]. Although the clinical results have improved dramatically, initial graft dysfunction still occurs in a significant number of liver recipients [2], [3]. Early graft failure remains a serious concern because it is associated with higher morbidity and early retransplantation rates, the latter significantly affecting long-term survival [4].

The ability of a graft to recover normal functions after reperfusion depends on its intrinsic quality before procurement (i.e. donor's factors), on the clinical status of the recipient, on the implantation period and more importantly on the preservation conditions. Ischemia and subsequent reperfusion are unavoidable causes of graft injury in the current practice of organ transplantation and represent a major risk factor for graft dysfunction [5], [6]. This multifactorial and interdependent damage process has become even more significant with increasing use of marginal grafts to meet the current growing demands [7]. Hence, developing strategies to minimize ischemia/reperfusion (I/R) injury could potentially have an enormous impact on transplant outcomes and support safer use of less than ideal organs [8].

In liver transplantation, the detrimental effects of I/R are mainly due to the acute generation of reactive oxygen species (ROS) following the reoxygenation process at time of revascularization [9]. Although low concentrations of ROS have an important role as mediators in normal cellular metabolism and signal transduction [10], in higher concentrations they are responsible for tissue damage. Indeed, overproduction of reactive molecules can initiate a cascade of deleterious cellular responses leading to inflammation, cell death and eventually organ failure [9]. A complex endogenous defense network, called the antioxidant system, has been developed in mammals to keep these hazardous products at a tolerable level. During a severe ischemic insult, this natural defense mechanism is however overwhelmed by the large amounts of ROS produced.

A large number of membrane and extracellular antioxidant agents have been tested experimentally to counterbalance the endogenous enzymatic depletion and improve liver graft initial function [11]–[15]. Other strategies used intracellular antioxidants components with controversial results [10], [16]–[18]. Overall, none of these strategies have found the way into routine clinical practice. Hence, the use of antioxidant agents with a long half-life and targeting both intra and extracellular sites might be more efficient to protect against the oxidative stress that is imposed to the liver allograft during the transplantation procedure.

Mangafodipir trisodium (MnDPDP), a magnetic resonance imaging contrast agent, is currently used for liver imaging, especially for the detection of liver neoplasm. Recently, it has been demonstrated that this molecule was endowed with powerful antioxidant properties with a high level of liver intracellular penetration [19]–[22]. *In vitro*, MnDPDP displays superoxide dismutase-like, catalase-like and glutathione reductase-like activities, allowing both detoxification of mitochondrial ROS and regeneration of the GSH pool [19]. Preventive administration of MnDPDP was associated with significant liver injury decrease in a mouse model of acetaminophen-induced acute liver failure [19]. More recently, the same group confirmed the protective properties of MnDPDP using a mouse model of hepatic I/R injury [23]. Besides, it has been reported that MnDPDP decreases myocardial damage in ischemic porcine myocardium [20]–[22]. In this case, MnDPDP improved the contractile function and reduced enzyme release in rat heart tissues during reoxygenation [20]. Another study showed that MnDPDP prevented myocardial cell damages through modulation of lysosomal and mitochondrial pathways [24]. However, the cellular mechanisms behind the protective effect of MnDPDP in the liver are currently unknown.

In the present study, by using an isolated perfused rat liver model, we demonstrated that MnDPDP administration to the donor shortly before liver harvesting also exerts a beneficial effect against cold I/R injury. Donor pretreatment leads to the maintenance of liver energetic status, attenuation of oxidative stress, mitochondrial damage and apoptosis, as well as an enhancement of nitric oxide production. This protective effect is also associated with the activation of Nrf2 and HIF-1α pathways.

## Materials and Methods

### Chemicals

Mangafodipir (MnDPDP), also marketed as Teslascan®, was supplied from Nycomed Amersham Health (Amersham, UK). Celsior® preservation solution was purchased from Genzyme (Saint Germain en Laye, France). All chemicals were from Sigma (Saint Quentin, Fallavier, France).

### Ethics Statement

Experiments were conducted according to the European Union regulations (Directive 86/609 EEC) for animal experiments and complied with our institution's guidelines for animal care and handling. Animal studies described herein were reviewered and approved (agreement no. 35–16 delivered to Dr. Anne Corlu, 06/08/2010) by the Directeur départemental des Services Vétérinaires de la Préfecture d'Ille et Vilaine (Rennes, France). Surgical procedure was conducted under general anesthesia and all animals were sacrificed by exsanguination upon liver procurement.

### Animals and Experimental groups

In this study, we used an isolated perfused rat liver model which is a useful experimental system for evaluating hepatic functions without the influence of other organ systems, undefined plasma constituents and neural-hormonal effects. Moreover, hepatic architecture, microcirculation and bile production are well preserved in this experimental model [13], [25].

Adult male Sprague-Dawley rats weighing 150 to 200 g (Janvier, Le Genest-St-Isle, France) were used in this study. Animals had ad libitum access to water and a standard diet. Rats were divided randomly into three groups: i) Control group (control, n = 6), ii) ischemia/reperfusion group (I/R, n = 6) and iii) MnDPDP+ischemia/reperfusion group (MnDPDP+I/R, n = 6 for each tested dose). In control group, livers were harvested from untreated rats, flushed with Ringer's lactate solution and immediately connected (without cold storage) via the portal vein to a recirculating perfusion system for 120 min at 37°C. Rats from I/R group received 1 ml of sodium chloride solution intraperitonealy 20 minutes prior to liver harvesting. After 24 hours of cold storage in Celsior® solution, livers were flushed with Ringer's lactate solution and immediately connected via the portal vein to a recirculating perfusion system for 120 min at 37°C. Rats belonging to MnDPDP+I/R group received 1 ml of MnDPDP intraperitonealy 20 minutes prior to liver harvesting. Three doses of MnDPDP were tested in this study (3, 5 and 15 µmol/kg body weight). The choice of MnDPDP concentrations was based on clinical studies showing that 5 µmol/kg corresponded to the relevant and safe dose for liver magnetic resonance imaging [26]. After 24 hours of cold storage in Celsior® solution, livers were flushed with Ringer's lactate solution and immediately connected via the portal vein to a recirculating perfusion system for 120 min at 37°C.

Twelve additional rats were used to evaluate tissue content of adenosine triphosphate (ATP) prior to *ex vivo* perfusion. These rats were divided randomly into three groups: i) normal liver group (n = 4): livers were harvested from untreated rats, ii) ischemia group (n = 4): 20 minutes after intraperitoneal administration of 1 ml of sterile sodium chloride solution, livers were harvested and subsequently preserved for 24 h at 4°C in Celsior® solution and iii) MnDPDP+Ischemia group (n = 4): 20 minutes after intraperitoneal administration of 1 ml of 5 µmol/kg MnDPDP, livers were harvested and subsequently preserved for 24 h at 4°C in Celsior® solution. Fragments of liver tissue were collected, frozen in liquid nitrogen and stored at −80°C until determination of adenosine triphosphate (ATP). This determination was only performed with the effective dose of MnDPDP.

### Liver procurement

Animals were anesthetized with an intraperitoneal injection of a mixture of Ketamine (90 mg/kg) and Xylasine (10 mg/kg). The surgical technique was performed as previously reported [13], [27]. Briefly, the liver was exposed through a midline incision. The common bile duct was cannulated distally with a polyethylene tube (PE n°50, ID 0.58 mm, Harvard Apparatus). The portal vein was isolated and the splenic and gastroduodenal veins were ligated. The infrarenal aorta was dissected free and cannulated (BD Insyte-W®, 20G, Le Pont-de-Claix, France). Then, the supra-celiac aorta was immediately cross-clamped, the suprahepatic vena cava was cut off after incision of the diaphragm. The organs were washed out via the infrarenal aorta with the ice-cold Celsior® solution (40 ml).

Once the liver was rinsed, the portal vein was cannulated (BD Insyte-W®, 16G, Le Pont-de-Claix, France). Liver washout was then completed through the portal vein (20 ml). Finally, the liver was retrieved carefully and stored for 24 hours, floating in 100 ml of Celsior® solution at 4°C.

### Perfusion of isolated liver

After 24 hours of cold preservation at 4°C, livers were flushed with lactated Ringer's solution through the portal vein. The aliquots of the effluent flush were sampled for measurements of cumulative alanine aminotransferase (ALT), aspartate aminotransferase (AST) and lactate dehydrogenase (LDH) after prolonged ischemia. In order to account for the period of rewarming during surgical implantation *in vivo*, all livers, except those of the control group, were exposed to room temperature (22°C) on a Petri dish for 30 minutes prior to reperfusion [13].

Livers were then immersed in the Krebs Henseleit Buffer (Sigma Aldrich, St Quentin Fallavier, France) in a perfusate chamber and connected via the portal vein to a continuous and closed perfusion circuit built in our laboratory [28]. Livers were reperfused for 120 min at 37°C. Time 0 corresponded to the satisfactorily connection of the portal catheter to the circuit. The perfusate was propelled in the circuit with a peristaltic pump (BVP-pump, Ismatec, Zurich, Switzerland). During the first 15 minutes of perfusion (initial equilibration period), the flow was gradually increased to reach a constant value of 3 ml/min/g of liver [25]. After the initial equilibration period, perfusion pressure was continuously monitored throughout the reperfusion period [13], [25]. The perfusate temperature (37°C) was regulated by means of a thermostatically controlled bath into which the perfusate chamber was placed [13], [29]. The buffer was continuously ventilated with a 95% O_2_ and 5% CO_2_ gas mixture and passed through a bubble trap prior to entering the liver [13], [28].

During the 120 min of normothermic reperfusion, samples were collected from the hepatic effluent fluid at 30 min intervals and stored at −80°C for later analyses. Bile was collected by gravity in graduated test tubes placed outside the thermostatically controlled bath to avoid dilution of the bile by condensed water. Bile output was determined at 120 min of reperfusion. At the end of the reperfusion period, fragments of liver tissue were collected, frozen in liquid nitrogen and stored at −80°C for further determinations. Some tissue samples were placed in paraformaldehyde for histological analyses.

### Bile output

Liver function was assessed by measurement of bile production [13]. Bile was collected through the cannulated bile duct and output reported in µl/120 min/g liver.

### Biochemical Determinations

Hepatic injury was assessed by determination of AST, ALT, LDH and gamma-GT levels in perfusate samples [13], [29] using an automatic analyzer (Olympus automated chemistry Analyzer AU 2700). Liver function was evaluated by measurement of albumin, alkaline phosphatase levels in perfusate samples, as well as by determination of biliary bilirubin excretion using an automatic analyzer (Olympus automated chemistry Analyzer AU 2700). Hepatic ATP content after cold ischemia, as well as after normothermic reperfusion was measured by using the bioluminescence assay kit from Sigma Aldrich (St Quentin Fallavier, France).

Lipid peroxidation was used as an indirect measurement of the oxidative stress induced by ROS [13]. It was determined in whole liver homogenates by measuring the formation of malondialdehyde (MDA) with the thiobarbiturate reaction [13], using the TBARS assay kit from Interchim (Montluçon, France). Hepatic catalase activity was determined by using the peroxidation function of catalase. This colorimetric method is based on the reaction of catalase with methanol in presence of sufficient concentration of hydrogen peroxide (Catalase assay kit from Interchim, Montluçon, France). SOD activity was measured in liver tissues with a method utilizing a tetrazolium salt for detection of superoxide radicals generated by xanthine oxidase and hypoxanthine. One unit of SOD is defined as the amount of enzyme needed to exhibit 50% dismutation of superoxide radical (SOD Activity Detection kit from Interchim, Montluçon, France). Purine Nucleotide Phosphorylase (PNP) activity was measured by an enzymatic method described by Nakagami et al [30]. Nitric Oxide (NO) production in liver was determined by tissue accumulation of nitrite and nitrate, as previously reported [13]. Activity of glutamate dehydrogenase (GLDH), a mitochondrial enzyme that indicates mitochondrial damages, was measured using an enzymatic method as previously described [13], [30].

### Western Blotting

Liver tissues were homogenized and subcellular fractions were prepared as previously described [13], [31]. Total liver lysates were used to quantify heme oxygenase 1 (HO1), constitutive NO synthase (eNOS), inducible NO synthase (iNOS), cytochrome c, caspase 9 and caspase 3 (total and cleaved) by Western blot. Cytosolic fractions were used to quantify cytochrome c and cleaved caspase 9. Mitochondrial fractions were used to determine the cytochrome c release. Nuclear fractions were used to determine hypoxia inducible factor-1α (HIF-1α) and nuclear factor-E2-related factor-2 (Nrf2). Proteins were separated by sodium dodecyl sulfate polyacrylamide gel electrophoresis and transferred to polyvinylidene fluoride membranes. Membranes were immunoblotted with antibodies directed against HSC70, HO1, Nrf2 and HIF-1α (Santa Cruz Biotechnology, Santa Cruz, CA), cleaved caspase 3, cleaved caspase 9,and cytochrome c (Cell Signalling Technology Inc., Beverly, MA), and against eNOS and iNOS (Transduction Laboratories, Lexington, KY). Protein expression was visualized using an enhanced chemiluminescence kit (ECF, Amersham Biosciences, Orsay, France). The values were obtained by densitometric scanning and the quantity software (Bio-Rad Laboratories, Hercules, CA, USA). The scanning values for HO1, eNOS, iNOS, cytochrome c, cleaved caspase 9 and 3, as well as HIF-1α and Nrf2 were normalized with the scanning values for HSC70, While, those for cytosolic and mitochondrial cytochrome c were normalized by the total cytochrome c. Cleaved caspase 3 and 9 were divided by the full length fragment of each protein, respectively.

### Histology and TUNEL Assay

For standard histology evaluation, a hematoxylin/eosin staining was performed [31]. Damage score were estimated by counting morphological alterations in 10 randomly selected microscopic fields from 6 samples of each group and from at least 3 independent experiments. The morphological liver integrity was graded on a scale of 1 (excellent) to 5 (poor). Grading was adapted from t'Hart et al [32] and described as: (1) normal rectangular structure, (2) rounded hepatocytes with an increase of the sinusoidal spaces, (3) vacuolization, (4) nuclear picnosis and (5) necrosis. Apoptosis was assessed by terminal deoxynucleotidyl transferase-mediated dUTP biotin nick end labeling (TUNEL, Roche Molecular Biochemical's, Mannheim, Germany). TUNEL-positive nuclei were counted in 10 random high power fields (×40 objectives) [31]. The apoptotic index was calculated as a ratio of the apoptotic cell number to the total cell number in each field and was expressed as a percentage. Histological evaluation of damage score and ratio of the apoptotic cell number were performed in a blinded fashion by 3 different observers.

### Statistical Analysis

Data were expressed as means ± SEM. Data were analyzed using Graph Pad Prism software (Version 4.0; Graph Pad, San Diego, CA). Statistical analysis was performed by variance analysis (Kruskal-Wallis test), followed by Mann-Whitney U test when the overall comparison of groups was significant. p<0.05 was considered as significant.

## Results

### MnDPDP protects against hepatic damage and maintains liver function

**Figure 1:**
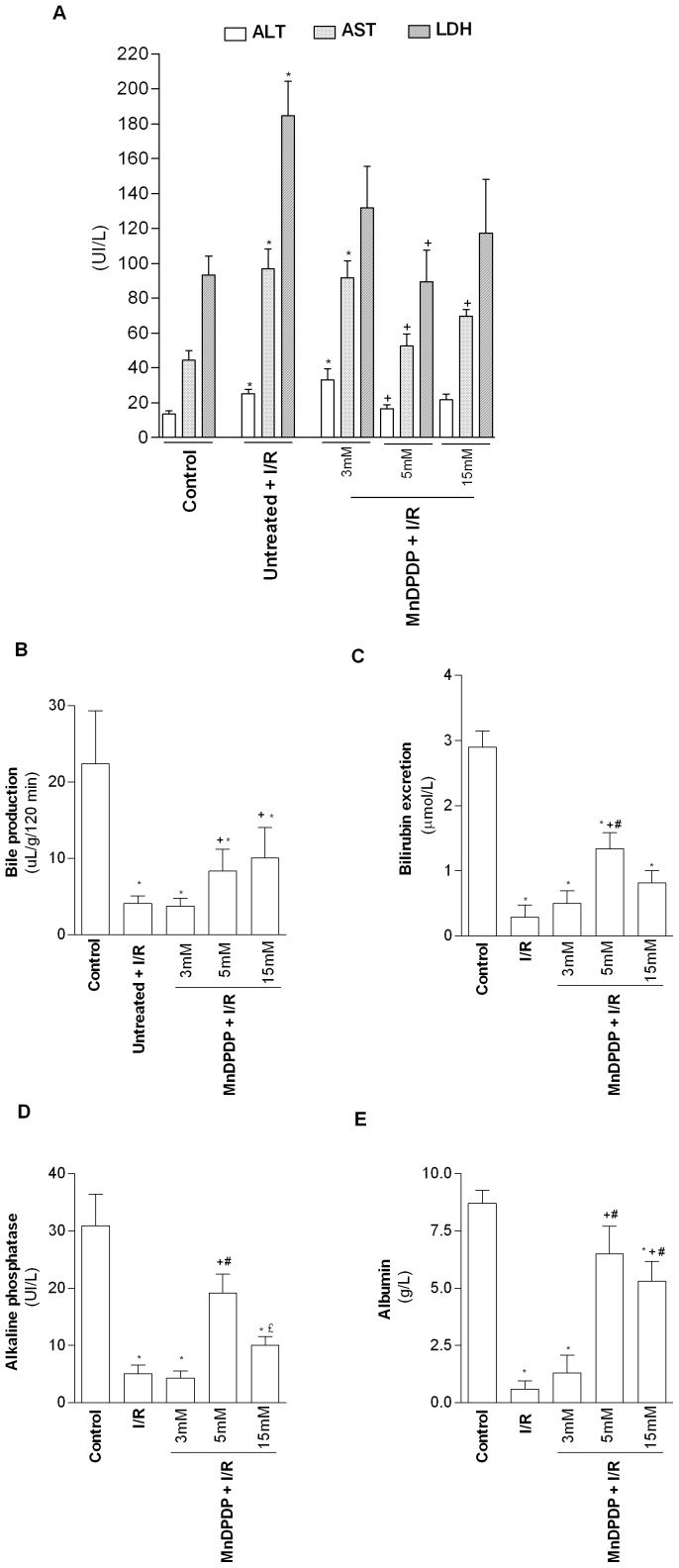
Mangafodipir limits the enzyme leakage and improve liver function. (A) Alanine amino transferase (ALT), Aspartate amino transferase (AST) and Lactate dehydrogenase (LDH) levels were measured in liver perfusates at the end of normothermic reperfusion (T = 120 min). (B) Bile production was assessed in livers along the 120 min of normothermic reperfusion. Biliary Bilirubin excretion (C) perfusate Albumin (D) and alkaline phosphatase (E) levels were determined at the end of normothermic reperfusion. Control group: untreated rat livers immediately submitted to 120 min of normothermic reperfusion; I/R group: liver procured from vehicle (NaCl) pretreated rats, cold stored for 24 h at 4°C and submitted to 120 min of normothermic reperfusion; MnDPDP+I/R group: livers procured from 3, 5 or 15 µmol/kg MnDPDP-pretreated rats, cold stored for 24 h at 4°C and submitted to 120 min of normothermic reperfusion. Results were expressed in UI/L. * *p*<0.05: either I/R or MnDPDP+I/R versus Control; ^+^ *p*<0.05: MnDPDP+I/R versus I/R. £ *p*<0.05: MnDPDP+I/R 15 µmol/kg versus 5 µmol/kg.

After 24 h of cold ischemia and 120 min of normothermic reperfusion, higher levels of enzyme leakage (ALT, AST and LDH ) were observed in cold stored livers (I/R group) compared with livers immediately perfused without cold preservation (control group) confirming the high sensitivity of parenchymal cells to cold ischemia and subsequent reperfusion (Figure 1 A). LDH and ALT levels in livers of rats pretreated with MnDPDP at a concentration of 3 µmol/kg and 15 µmol/kg were similar to those observed in I/R group. In contrast, pretreatment with MnDPDP at 5 µmol/kg caused a significant reduction of ALT, AST and LDH activities when compared to I/R group (Figure 1 A). Similar patterns for ALT, AST and LDH were observed at 30, 60 and 90 min (data not shown). Noteworthy, no statistical difference was found for cumulative ALT, AST and LDH release in flushing effluent between I/R group and MnDPDP-treated group at the end of cold ischemia (data not shown). In all experimental conditions, Gamma GT levels were below to the threshold detection. Importantly, the MnDPDP dose-effect relationship was correlated with hepatocellular functional recovery upon reperfusion as indicated by higher bile production, biliary bilirubin excretion, albumin and alkaline phosphatase production in pretreated rats with MnDPDP at 5 µmol/kg or 15 µmol/kg compared to I/R group (Figure 1). MnDPDP administration at 3 µmol/kg did not improve liver function. Based on hepatocellular damage and functional parameters, the maximal protective effect of MnDPDP was obtained at the dose of 5 µmol/kg.

**Figure 2:**
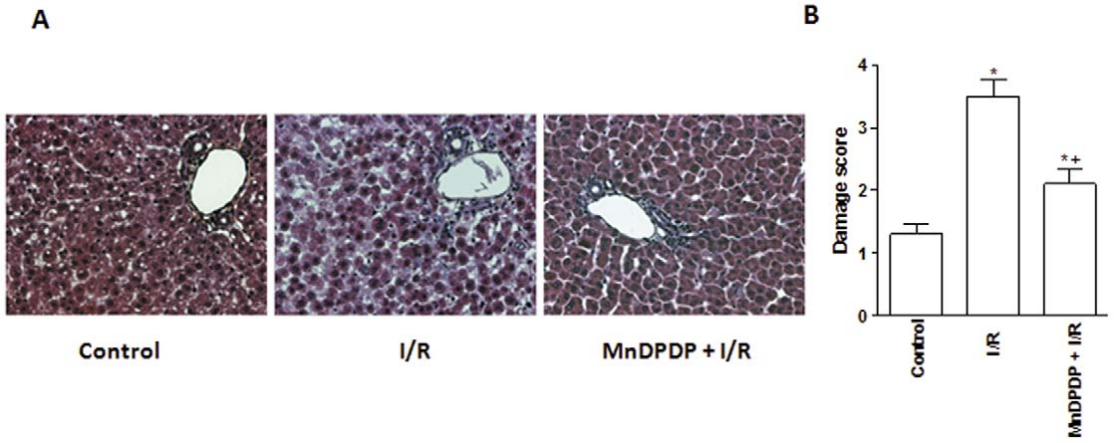
Mangafodipir prevents histological lesions. Hematoxylin eosin stained sections were used for the evaluation of histological lesions (A) (magnification ×40) and damage score (B) in livers harvested from untreated rats and immediately submitted to 120 min of normothermic reperfusion (Control group), in livers procured from vehicle (NaCl) pretreated rats, cold stored for 24 h at 4°C and submitted to 120 min of normothermic reperfusion (I/R group) and in livers procured from 5 µmol/kg MnDPDP-treated rats, cold stored for 24 h at 4°C and submitted to 120 min of normothermic reperfusion (MnDPDP+I/R group). * *p*<0.05: either I/R or MnDPDP+I/R versus Control; ^+^ *p*<0.05: MnDPDP+I/R versus I/R.

Histological changes following I/R were in keeping with biochemical parameters of hepatic injury and only histological findings with the most effective dose of MnDPDP (5 µmol/kg) are presented in figure 2. Standard histological examination revealed pronounced hepatocyte vacuolization and marked disintegration of hepatic architecture in I/R group as compared to control group (Figure 2A), the damage score being significantly different between the two groups (3.5±0.2 *vs* 1.2±0.1, *p*<0.05) (figure 2B). Hepatocyte integrity was better maintained in 5 µmol/kg MnDPDP pretreated livers than in I/R group. Hepatocyte in MnDPDP+I/R group appeared at most rounded with an increase of the sinusoidal spaces, with a damage score significantly lower relative to I/R group (2.1±0.2 *vs* 3.5±0.2, *p*<0.05) (Figure 2). Altogether, these observations led us to evaluate the mechanisms by which MnDPDP protects livers against the deleterious effects of I/R injury only at the effective dose of 5 µmol/kg.

### MnDPDP preserves liver energy status following cold preservation and normothermic reperfusion

**Figure 3:**
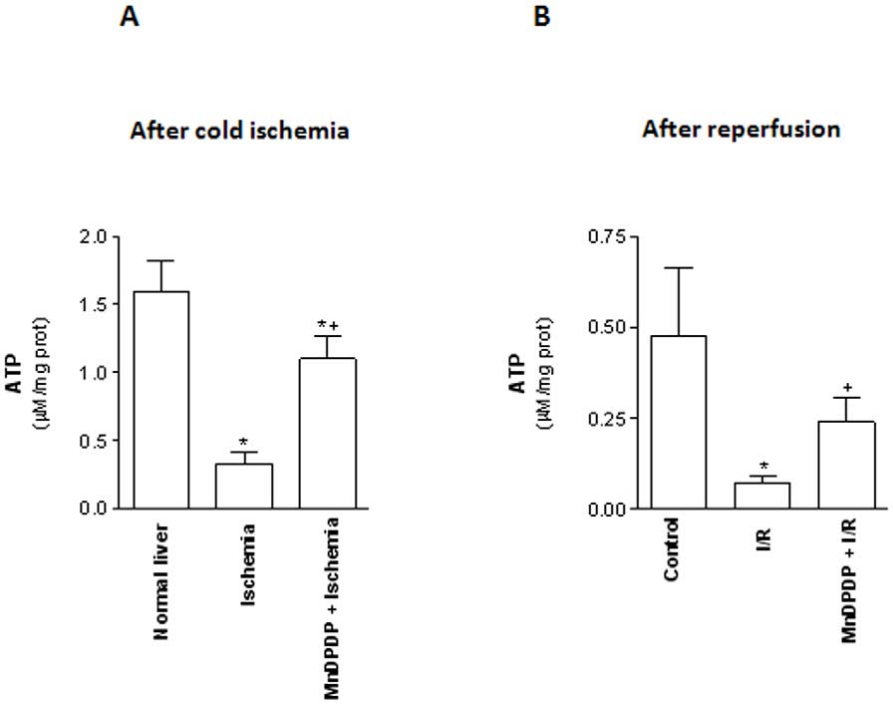
Mangafodipir prevents ATP depletion. (A) Hepatic ATP content were measured in normal livers, in livers procured from sodium chloride solution pretreated rats and cold stored for 24 h at 4°C (Ischemia group) and in livers procured from 5 µmol/kg MnDPDP-treated rats and cold stored for 24 h at 4°C (MnDPDP+Ischemia group). Results were expressed as µmol/mg liver. * *p*<0.05, either Ischemia or MnDPDP+Ischemia versus Control; ^+^ *p*<0.05, MnDPDP+Ischemia versus Ischemia. (B) Hepatic ATP content were measured in livers harvested from untreated rats and immediately submitted to 120 min of normothermic reperfusion (Control group), in livers procured from vehicle (NaCl) pretreated rats, cold stored for 24 h at 4°C and submitted to 120 min of normothermic reperfusion (I/R group) and in livers procured from 5 µmol/kg MnDPDP-treated rats, cold stored for 24 h at 4°C and submitted to 120 min of normothermic reperfusion (MnDPDP+I/R group). * *p*<0.05: either I/R or MnDPDP+I/R versus Control; ^+^ *p*<0.05: MnDPDP+I/R versus I/R.

As shown in figure 3A, lower amounts of ATP were observed in 24 h-cold stored livers (Ischemia group) when compared to non-preserved livers (normal livers). Similarly, ATP recovery after 120 min normothermic reperfusion was lower in livers that had been cold stored for 24 h in celsior® solution (I/R group) compared to control group (figure 3B).

In contrast, depletion of ATP content was significantly attenuated in MnDPDP pretreated livers at the end of cold preservation relative to ischemia group (1.10±0.16 µmol/mg liver *vs* 0.32±0.09 µmol/mg liver, *p*<0.05) (Figure 3A). Also, MnDPDP pretreatment improved ATP recovery at the end of the normothermic reperfusion period compared with I/R group (0.23±0.06 *vs* 0.07±0.02 µmol/mg liver, *p*<0.05) (Figure 3B).

### MnDPDP protects against hepatic apoptosis and mitochondrial injury

**Figure 4:**
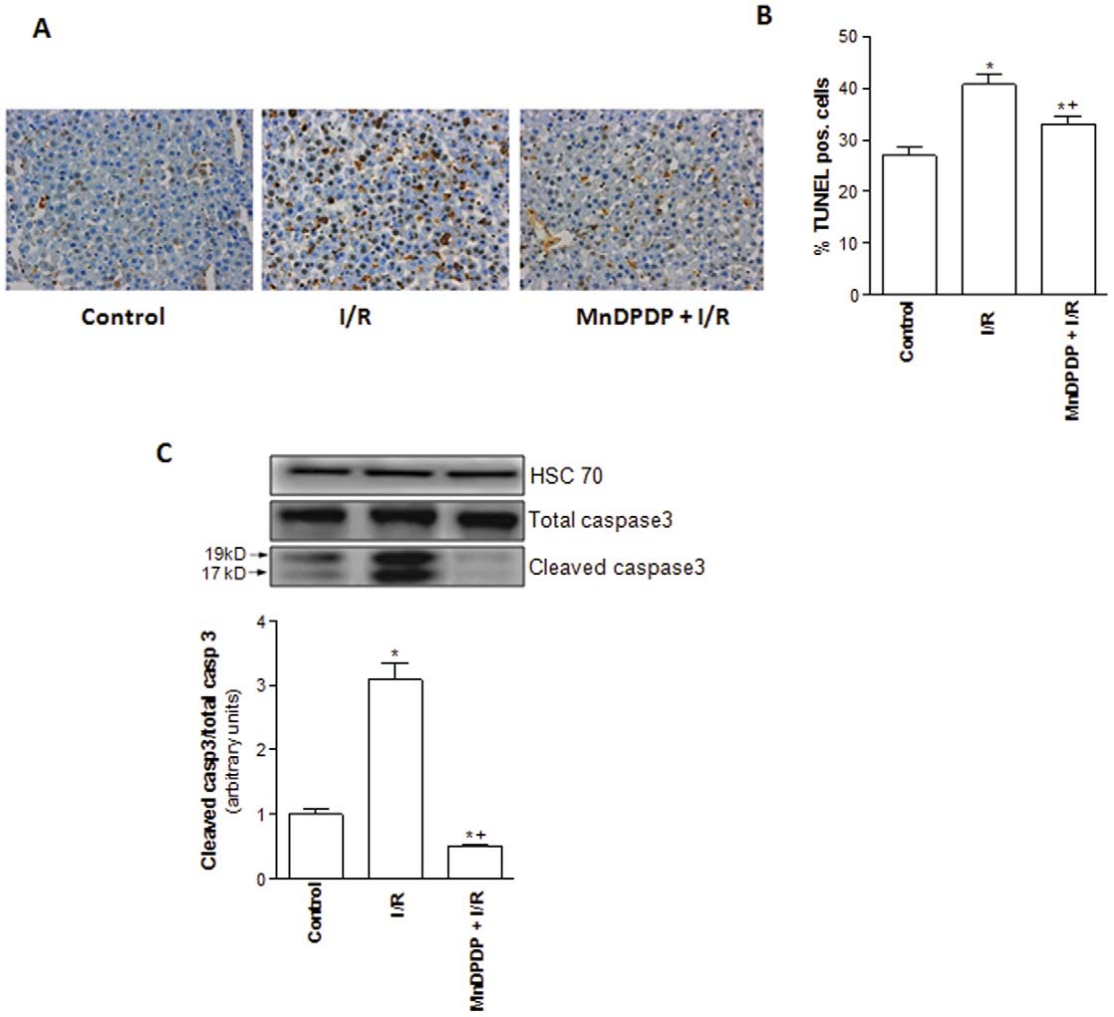
Mangafodipir prevents liver cells apoptosis. (A) TUNEL staining of livers (original magnification ×40) and (B) Percentage of TUNEL-positive hepatocytes was evaluated in livers harvested from untreated rats and immediately submitted to 120 min of normothermic reperfusion (Control group), in livers procured from vehicle (NaCl) pretreated rats, cold stored for 24 h at 4°C and submitted to 120 min of normothermic reperfusion (I/R group) and in livers procured from 5 µmol/kg MnDPDP-treated rats, cold stored for 24 h at 4°C and submitted to 120 min of normothermic reperfusion (MnDPDP+I/R group). * *p*<0.05: either I/R or MnDPDP+I/R versus Control; ^+^ *p*<0.05: MnDPDP+I/R versus I/R. (C) Western blot and densitometry analysis of cleaved caspase-3 protein in livers from control, I/R and MnDPDP+I/R groups. Western blot data represent one of three independent experiments with similar results. Western blot densitometry data are normalized for the loading control HSC70. * *p*<0.05: either I/R or MnDPDP+I/R versus Control; ^+^ *p*<0.05: MnDPDP+I/R versus I/R.

**Figure 5:**
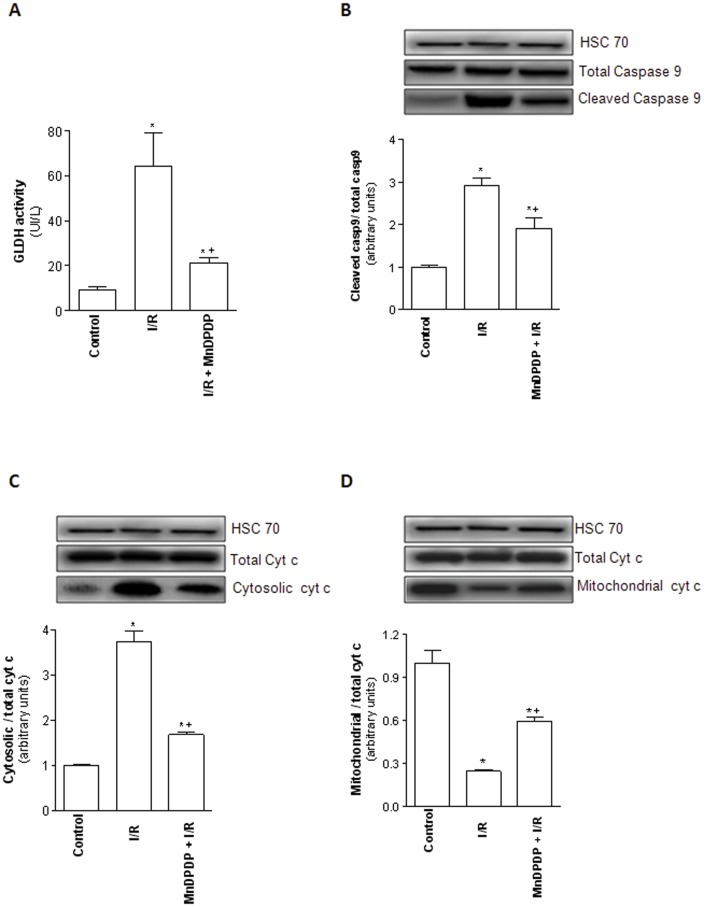
Mangafodipir reduces mitochondrial damage. (A) GLDH perfusate levels were measured in livers harvested from untreated rats and immediately submitted to 120 min of normothermic reperfusion (Control group), in livers procured from vehicle (NaCl) pretreated rats, cold stored for 24 h at 4°C and submitted to 120 min of normothermic reperfusion (I/R group) and in livers procured from 5 µmol/kg MnDPDP-treated rats, cold stored for 24 h at 4°C and submitted to 120 min of normothermic reperfusion (MnDPDP+I/R group). Results were expressed in UI/L. * *p*<0.05: either I/R or MnDPDP+I/R versus Control; ^+^ *p*<0.05: MnDPDP+I/R versus I/R. Western blot and densitometry analysis of cleaved caspase 9 (B) and cytochrome c in cytosol (C) and mitochondria (D) in livers from control, I/R and MnDPDP+I/R groups. Western blot data represent one of three independent experiments with similar results. Western blot densitometry data are normalized for the loading control HSC70. * *p*<0.05: either I/R or MnDPDP+I/R versus Control; ^+^ *p*<0.05: MnDPDP+I/R versus I/R.

Cold ischemia followed by 120 min of normothermic reperfusion was associated with an increased number of apoptotic liver cells as demonstrated by the higher percentage of TUNEL-positive hepatocytes when compared to control group (Figure 4A and B). This observation was correlated with an increased release of the cleaved forms of caspase-3 (19 and 17 kDa) in I/R group relative to controls (Figure 4C). Interestingly, a significant reduction in cell TUNEL staining and cleaved caspase-3 levels was evidenced when MnDPDP was administered to liver donors (Figure 4). To further evaluate the mechanisms leading to liver cell injuries, we also investigated mitochondrial damage. Cold ischemia followed by 120 min of normothermic reperfusion led to significant (*p*<0.05) increases of GLDH activity (Figure 5A), cleavage of caspase-9 (Figure 5B) and release of mitochondrial cytochrome c into cytoplasm (Figure 5C and D). MnDPDP pretreatment improved mitochondrial integrity as evidenced by the lower increase of GLDH activity, caspase-9 cleavage and cytochrome c release compared to the liver of I/R group (*p*<0.05).

### MnDPDP protects against oxidative stress

**Figure 6:**
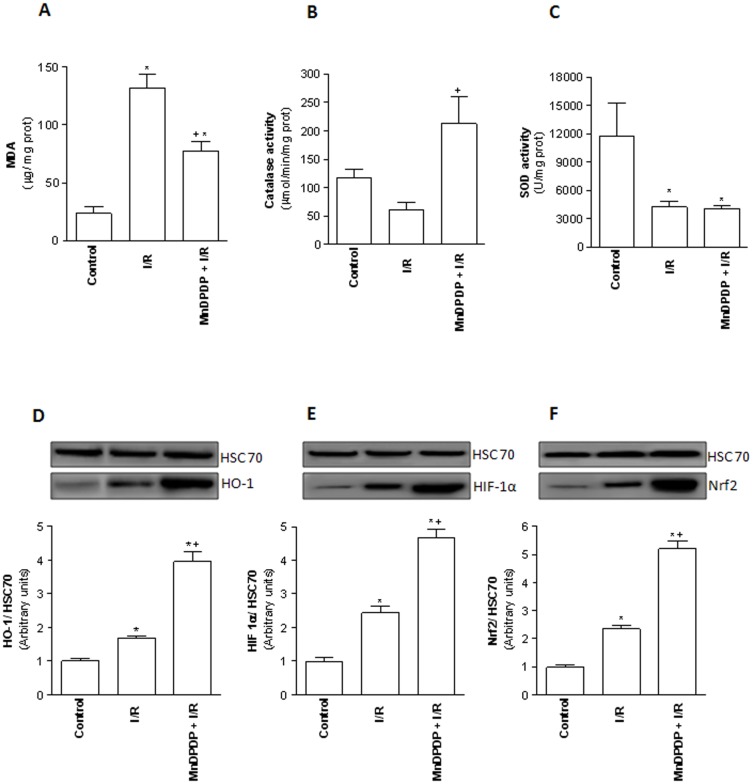
Mangafodipir prevents lipid peroxidation and oxidative stress. (A) Hepatic malondialdehyde (MDA) levels and Catalase (B) and superoxide dismutase (C) activities were measured in livers harvested from untreated rats and immediately submitted to 120 min of normothermic reperfusion (Control group), in livers procured from vehicle (NaCl) pretreated rats, cold stored for 24 h at 4°C and submitted to 120 min of normothermic reperfusion (I/R group) and in livers procured from 5 µmol/kg MnDPDP-treated rats, cold stored for 24 h at 4°C and submitted to 120 min of normothermic reperfusion (MnDPDP+I/R group). * *p*<0.05: either I/R or MnDPDP+I/R versus Control; ^+^ *p*<0.05: MnDPDP+I/R versus I/R. Western blot and densitometry analysis of HO-1 (D), HIF-1α (E), and Nrf2 (F) in livers from control, I/R and MnDPDP+I/R groups. Western blot data represent one of three independent experiments with similar results. Western blot densitometry data are normalized for the loading control HSC70. * *p*<0.05: either I/R or MnDPDP+I/R versus Control; ^+^ *p*<0.05: MnDPDP+I/R versus I/R.

Lipid peroxidation has been used as an indirect marker of ROS-induced hepatic damage. As shown in Figure 6A, MDA levels were significantly increased in I/R group when compared to control group (131±12.4 µg/mg protein *vs* 24±7 µg/mg protein, *p*<0.05). Increase of MDA levels in liver rats pretreated with MnDPDP was significantly reduced (77.8±7.9 µg/mg protein) compared to I/R group (131±12.4 µg/mg protein, *p*<0.05). Pretreated livers also exhibited a significant higher catalase activity (213±46.5 µmol/min/mg protein) when compared to values found in I/R group (61.7±12.6 µmol/min/mg protein) (Figure 6B). SOD activity level was similar between MnDPDP-pretreated and unpretreated group (Figure 6C). In parallel, rat pretreatment with MnDPDP significantly increased liver HO-1, HIF-1α and Nrf2 expressions compared to observed levels in livers from I/R group (figure 6D–F).

### MnDPDP protects endothelial cells and favors nitric oxide production

**Figure 7:**
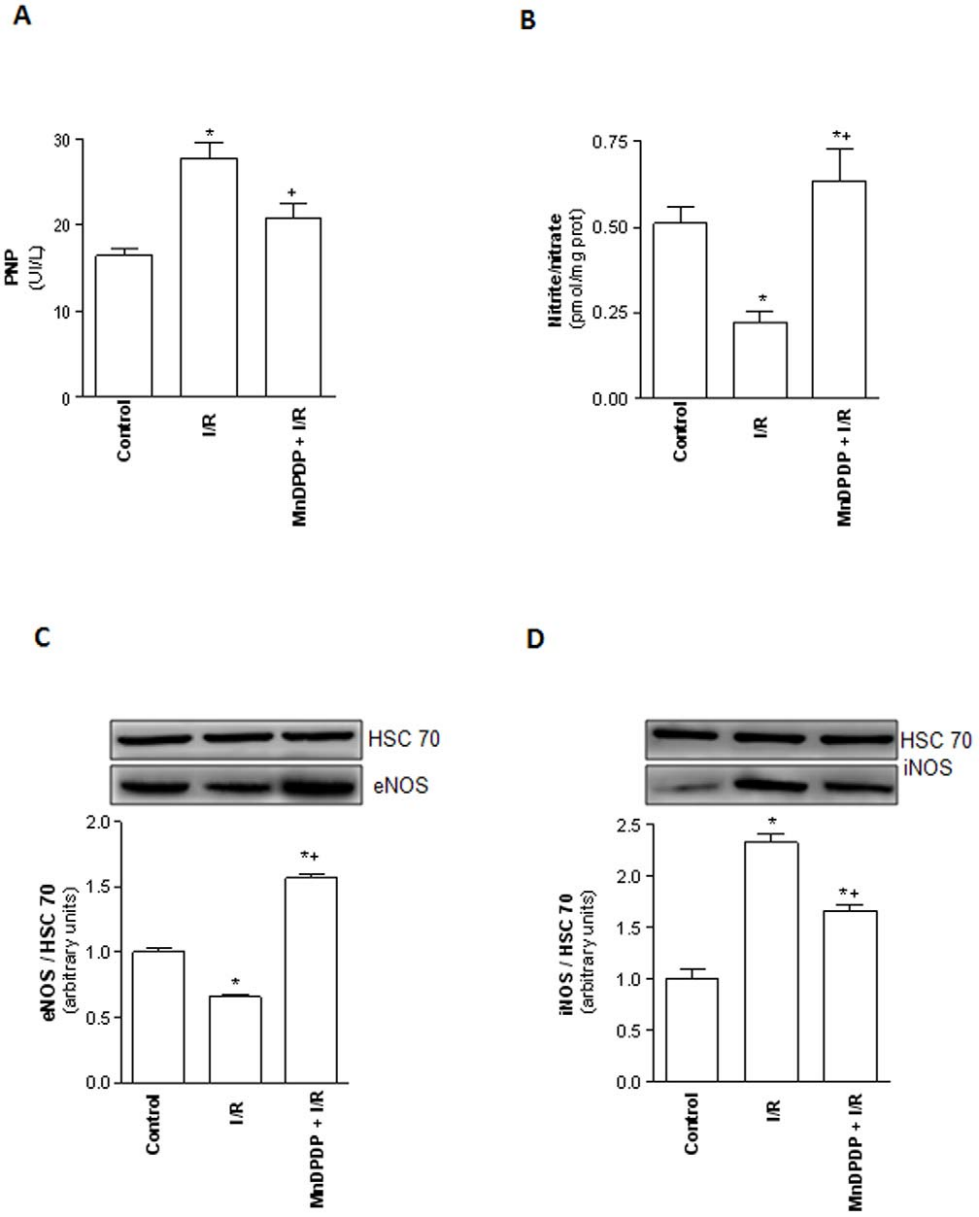
Mangafodipir limits endothelial cells injury and favors nitric oxide production. Perfusate Purine Nucleotide Phosphorylase (PNP) activity (A) and tissue accumulation of Nitrate/nitrite levels (B) were measured in livers harvested from untreated rats and immediately submitted to 120 min of normothermic reperfusion (Control group), in livers procured from vehicle (NaCl) pretreated rats, cold stored for 24 h at 4°C and submitted to 120 min of normothermic reperfusion (I/R group) and in livers procured from 5 µmol/kg MnDPDP-treated rats, cold stored for 24 h at 4°C and submitted to 120 min of normothermic reperfusion (MnDPDP+I/R group). Western blot and densitometry analysis of constitutive nitric oxide synthase (eNOS) (C) and inducible nitric oxide synthase (iNOS) (D) in livers from control, I/R and MnDPDP+I/R groups. Western blot data represent one of three independent experiments with similar results. Western blot densitometry data are normalized for the loading control HSC70. * *p*<0.05: either I/R or MnDPDP+I/R versus Control; ^+^ *p*<0.05: MnDPDP+I/R versus I/R.

ROS injury to the endothelial cells was detected by quantification of PNP. As shown in figure 7A, rat livers from I/R group released significant higher levels of PNP into the perfusate compared with control group. MnDPDP pretreatment (MnDPDP+I/R group) partially prevented PNP release relative to I/R group (Figure 7A). The effect of MnDPDP on Nitric Oxide (NO) production which reflects the nitrite and nitrate contents in liver tissues was also evaluated. As expected, hepatic NO level was decreased in I/R group compared to the control group (Figure 7B). By contrast, a higher level of NO production was observed in livers of rats pretreated with MnDPDP in comparison to levels observed in I/R group. This increase in NO production was well correlated with the constitutive NO synthase (eNOS) expression level (Figures 7C). Conversely, the expression of inducible NO synthase (iNOS) was induced in livers of I/R group compared to control group and MnDPDP pretreatment partially prevented this increase (Figure 7D).

## Discussion

Ample evidence suggests that the generation of ROS is critical in the pathogenesis of I/R injury [9]. Despite the mainly vascular origin of the post-ischemic oxidant stress, ROS generated by Kupffer cells or adherent neutrophils is responsible for a substantial intracellular oxidant stress in hepatocytes. This suggests that intracellular defense mechanisms are critical for detoxification of ROS generated by intracellular as well as extracellular sources [31]–[33].

In this study, using an isolated perfused rat liver model, we demonstrate for the first time that MnDPDP protects the rat liver from cold I/R injury when administrated to the donor prior to liver harvesting. MnDPDP pretreatment at concentration of 5 µmol/kg improved graft function, lessened hepatocellular as well as endothelial injury and allowed a better-preserved liver integrity. This protective effect was associated with attenuation of oxidative stress, mitochondrial damage and apoptosis. These observations are of interest since the efficient concentration of 5 µmol/kg corresponded to the relevant and safe dose used in the clinical setting for liver magnetic resonance imaging [26]. Moreover, MnDPDP pharmacogical and pharmacokinetic properties favor high level of liver intracellular penetration. This property is related to the fodipir moiety of MnDPDP, a chelate of manganese resulting from the condensation of two pyridoxal 5′-phosphate molecules [21]. This fodipir moiety binds to the pyridoxal 5′-phosphate receptor on hepatocytes and ensures a high intrahepatic concentration of MnDPDP through its specific biding to vitamin B6 receptors expressed by hepatocytes [23].

The role of the cell membrane is to maintain the cell structure and communication lines to the extracellular compartment. The release of intracellular enzymes into the perfusate closely reflects the leakiness of the cell membrane and, therefore, indirectly predicts the degree of organ damage [34], [35]. A study on transmembranous ion exchange by means of magnetic resonance spectroscopy has also shown that injury of the hepatic cell membrane play an important role in graft dysfunction [36]. In our study, pretreatment with 5 µmol/kg of MnDPDP reduced the release of transaminases and LDH in the course of normothermic reperfusion and was also associated with a better histological integrity, suggesting lower parenchymal injury.

Accordingly, MnDPDP administration also led to a greater capacity of bile production relative to untreated rats and correlated to the lesser hepatic damage. In the isolated perfused liver model, bile production is widely considered as a reliable marker of global liver function [25], [34]. It is a complex process that requires both the preservation of the ultrastructural, biochemical and physiological integrity of the liver [34]. It is noteworthy that bile secretion is an active mechanism that closely rely on Na+/K+ ATPase activity [37], indirectly reflecting the ability of hepatocytes to synthesize ATP [38]. ATP represents an indispensable energy source to the cell for the maintenance of its structural and functional integrity. It has been shown that ATP depletion in hepatocytes leads to an increase in cytolytic protease activity [39]. In our study, we showed that improvement of post-ischemic bile flow in MnDPDP-pretreated livers was consistently associated with higher tissue ATP content after cold ischemia, as well as after normothermic reperfusion. Indeed, MnDPDP administration limited the depletion of ATP reserves after cold ischemia as compared to untreated animals. In the clinical setting, it has been shown that liver ATP content before implantation was an independent predictor of initial graft function [9], [40], [41].The potential of MnDPDP to enhance liver energy metabolism may be related to its efficiency in carrying and releasing manganese ions to the hepatocytes in a time shorter than the plasmatic clearance. Manganese is an essential element required in living organisms both as an activator and a constituent of several enzymes. In addition, the active form of ATP implies the presence of magnesium and manganese. Manganese is a cofactor of mitochondrial superoxide dismutase, an antioxidant enzyme that scavenges oxygen free radicals [42] and is involved in a major metabolic pathway such as the electron transport system. While cold storage by itself is known to induce Ca2+ uptake that causes an inhibition of mitochondrial ATP synthesis, it could be also expected that the presence of Mn2+ at low doses after MnDPDP dissociation may efficiently antagonize several adverse effects of Ca2+ on mitochondria.

A well-known mechanism of cell death induced by extracellular ROS is lipid peroxidation. Since polyunsaturated fatty acids in the cell membrane are vulnerable to lipid peroxidation, this free radical-mediated process leads to liver cell death very rapidly [43]. In our conditions, MnDPDP pretreatment prevented lipid peroxidation as indicated by significant lower MDA levels in MnDPDP-pretreated livers when compared to levels found in I/R group. This effect supports an antioxidant action of MnDPDP in the post-ischemic liver tissue. Using different models of stress, previous studies indicate that a protective effect of MnDPDP is associated with catalase-, SOD- and GSH reductase-like properties, allowing this compound to act at multiple steps of the ROS cascade [19], [20], [22], [23]. It has also been suggested that MnDPDP protects leukocytes from apoptosis induced either by H_2_O_2_ or by chemotherapeutic agents like oxaliplatin and paclitaxel [44], and prevent Huh-7 hepatoma cell death induced by H_2_O_2_ and superoxide anion [19]. In our conditions, there was no difference in SOD and GSH reductase activities (data not shown) between livers procured from MnDPDP-pretreated rats and unpretreated ones. However, MnDPDP-pretreated livers exhibited a higher catalase activity that may be responsible, at least in part, for the beneficial effects against oxidative stress. Interestingly, it has been demonstrated in animal models, that an increase of catalase activity significantly suppresses the subsequent I/R-induced acute liver injury [45]. Moreover, like various macromolecular substances, catalase represents a new class of therapeutic agents that has been examined for various ROS-mediated injuries, especially those associated with I/R [45]. In our conditions, the protection against oxidative stress might also be explained by the capacity of MnDPDP to increase the expression of Nrf2, HIF-1α and HO-1. On one hand, Nrf2 binds to the antioxidant response element in the promoter region of numerous genes encoding antioxidative and phase 2 enzymes, including HO-1, NAD(P)H: quinone oxidoreductase 1, glutathione reductase and glutathione peroxidase. Phase 2 enzymes play a major role in the detoxification of ROS during I/R [46]. On the other hand, HIF-1α triggers an increase in gene expression involved in glycolysis, glucose metabolism, mitochondrial function, cell survival and resistance to oxidative stress. Induction of HO-1 has also been implicated in numerous clinically relevant disease states and I/R injury [47]–[49]. It has been suggested that this enzyme exerts anti-oxidative, anti-inflammatory and anti-apoptotic functions [50]. Interestingly both transcriptional factors can activate HO-1. Overall, the protective effect of MnDPDP could be related to the activation of Nrf2 and HIF-1α pathways that lead to the induction of HO-1, catalase, glutathione reductase activities and improvement of liver functions.

The effectiveness of MnDPDP in reducing I/R injury possibly involves other undefined mechanisms. Sinusoidal endothelial cells (EC) have been described as the least tolerant non-parenchymal hepatic cells to I/R injury and represent the initial target of oxidative and inflammatory mediated damages [51]. Hepatic EC are particularly vulnerable to cold ischemia which induces serious morphological alterations such as retraction, cell body detachment, and sinusoidal denudation. EC dislocation is responsible for impaired graft microcirculation, platelet activation and adhesion molecules up-regulation. Cytoskeleton disorders also contribute to Kupffer cell activation, neutrophil infiltration, and finally hepatocyte death upon reperfusion. Moreover, there is a direct correlation between EC injury and length of cold ischemia, graft function and, above all, long term outcome after liver transplantation [52]. Notably, our present data suggest that pretreatment of donor with MnDPDP improved the tolerance of liver EC to I/R injury, as reflected by significant lower levels of PNP release into the perfusate. PNP being primarily localized in the cytoplasm of the endothelial cells, its release by the preserved livers indicates a lysis of those non-parenchymal cells. PNP release has been shown to be inversely related to sinusoidal EC viability and also reflect the oxidative stress intensity of livers subjected to I/R injury [52]. Although the mechanism behind this effect remains unclear, a direct EC protective effect by the MnDPDP and/or an indirect effect mediated through improved microcirculation are plausible.

MnDPDP was also described to stabilize and conserve nitric oxide (NO) generated in intact EC, thus protecting them against I/R injury [26], [53]. NO is produced from L-Arginine by nitric oxide synthases (NOS) that exists in both inducible (iNOS) and constitutive (eNOS) forms. A low concentration of NO produced by eNOS may serve to maximize blood perfusion, promote cell survival and protect liver against I/R injury [54] while a sustained expression of iNOS might become detrimental through an increase of toxic ROS, leading to liver injury [54], [55]. In the present study, we observed that MnDPDP increased total NO production, as evidenced by the nitrite/nitrate levels. This increase of NO levels could be beneficial in our condition since it was associated with a higher expression of eNOS and a lower expression of iNOS in MnDPDP-pretreated livers. Moreover, our results are in agreement with previous studies showing that the generation of ROS is needed along with NO to confer organ protection after ischemic preconditioning. In our condition, NO production after MnDPDP treatment was associated with a moderate ROS generation relative to controls.

To further elucidate the mechanisms by which MnDPDP conferred liver protection against I/R injury, we also evaluated apoptotic cell death and mitochondrial damage. Apoptosis has been identified as a hallmark of I/R-induced graft damage [56], [57]. The mitochondrial cytochrome c release into the cytosol is known as an early molecular event of apoptosis in response to oxidative load in the mitochondria [31], [58]. It further induces caspase 9 activation that in turn leads to the activation of the effector caspase 3, a downstream event in the apoptotic cascade [59]. Under our conditions, we could notice that MnDPDP pretreatment was associated with a decrease of hepatocyte apoptosis as evidenced by a significant reduction in the percentage of TUNEL positive hepatocytes, as well as a decrease of cleaved caspase-3 levels compared to those observed in untreated livers. In addition, the administration of MnDPDP to the donor improved mitochondrial integrity. Indeed, GLDH activity, cleaved caspase-9 level and cytosolic cytochrome c release were significantly reduced whereas mitochondrial cytochrome c amount was significantly increased when MnDPDP was administered to the donor prior to liver harvesting. Taken together, our data suggest that mitochondria might also be an important target through which MnDPDP exerts its cytoprotective effect. Our findings support previous data regarding protective effects of MnDPDP on cardiac dysfunction after reperfusion [22], [60] and liver injury [19], [23] through prevention of mitochondrial apoptotic cascade and caspase-3 activation.

In conclusion, donor pretreatment with MnDPDP is effective for protecting the liver from post-ischemic reperfusion injury. It confers protection through attenuation of ATP depletion, oxidative stress, apoptosis and endothelial cells as well as mitochondrial damages. This is the first study showing the potential interest of this molecule in the field of organ preservation. Since MnDPDP is safely used in liver imaging for many years, this preservation strategy holds great promise for translation to clinical liver transplantation. Further investigation in large animal model of liver transplantation will be required to elucidate the relevance of MnDPDP, for clinical liver transplantation.
